# Pulmonary high-resolution computed tomography findings in patients with synovitis, acne, pustulosis, hyperostosis and osteitis syndrome

**DOI:** 10.1371/journal.pone.0206858

**Published:** 2018-12-05

**Authors:** Chen Li, Shuang Liu, Xin Sui, Jian Wang, Wei Song, Wenshuai Xu, Kai-Feng Xu, Xinlun Tian, Wen Zhang

**Affiliations:** 1 Department of Traditional Chinese Medicine, Peking Union Medical College Hospital, Chinese Academy of Medical Sciences & Peking Union Medical College, Beijing, China; 2 Institute of Clinical Medicine, Peking Union Medical College Hospital, Chinese Academy of Medical Sciences & Peking Union Medical College, Beijing, China; 3 Department of Radiology, Peking Union Medical College Hospital, Chinese Academy of Medical Sciences & Peking Union Medical College, Beijing, China; 4 Department of Respiratory Medicine, Beijing Shunyi Airport Hospital, Beijing, China; 5 Department of Respiratory Medicine, Peking Union Medical College Hospital, Chinese Academy of Medical Sciences & Peking Union Medical College, Beijing, China; 6 Department of Rheumatology and Clinical Immunology, Peking Union Medical College Hospital, Chinese Academy of Medical Sciences & Peking Union Medical College, Beijing, China; University of Mississippi Medical Center, UNITED STATES

## Abstract

**Objective:**

To characterize the high-resolution computed tomography (HRCT) pulmonary abnormalities in patients with synovitis, acne, pustulosis, hyperostosis, and osteitis (SAPHO) syndrome.

**Methods:**

Pulmonary HRCT images were reconstructed from whole-spine computed tomography (CT) images of 67 patients with SAPHO syndrome. HRCT images of 58 healthy controls were also obtained and reviewed. Patients with pneumonia and tuberculosis were excluded. Demographic and clinical data such as gender, age, onset age, disease duration, erythrocyte sedimentation rate (ESR), highly sensitive C-reactive protein (hs-CRP) and the Bath Ankylosing Spondylitis Disease Activity Index (BASDAI) were collected from the SAPHO syndrome patients. Demographic characteristics, ESR and hs-CRP data from the healthy controls were also recorded. Student’s *t* test, Mann-Whitney U test, chi-squared test and logistic regression were employed to compare the HRCT findings of the two groups of patients.

**Results:**

The median age of the SAPHO syndrome patients was 47.0 years, interquartile range [38.0–53.0]; that of the healthy controls was 37.0[30.8–53.8]. From the detailed HRCT evaluations, abnormalities were identified in 45 patients. We found irregular linear opacities in 29 (43.3%) patients, opacities in 22 (32.8%), ground-glass opacity in 11 (16.4%), pleural thickening in 9 (13.4%), solitary nodules in 6 (9%), bronchiectasis in 3 (4.5%), pulmonary bulla in 2 (3%), multiple nodules in 1 (1.5%), and reticular patterns in 1 (1.5%). Compared to the healthy controls, the SAPHO syndrome patients had a significantly higher rate of opacities but a significantly lower percentage of nodules (especially multiple nodules), although the overall rates of abnormal HRCT findings were similar in the two groups. According to the multivariate logistic regression analysis, increased age and BASDAI < 4 were significant predictors of abnormal HRCT findings.

**Conclusion:**

Our study is the first to address HRCT pulmonary abnormalities in SAPHO syndrome patients. SAPHO syndrome patients have a significantly higher percentage of opacities and a significantly lower rate of pulmonary nodules than healthy controls. BASDAI and age are possible good predictors of abnormal HRCT pulmonary findings.

## Introduction

Synovitis, acne, pustulosis, hyperostosis and osteitis (SAPHO) syndrome is a spectrum of heterogeneous diseases, including osteoarticular and dermatological manifestations, first proposed by Chamot et al in 1987[1]. The core physiological changes that occur in SAPHO syndrome are osteitis and bone hypertrophy, which mainly manifest as pain and activity limitations in the affected area[2–4]. The major dermatological manifestations include palmoplantar pustulosis (PPP) and severe acne (SA), with psoriasis vulgaris considered a concomitant condition[5]. The pathogenesis of SAPHO syndrome has not yet been elucidated, and the means of early diagnosis and targeted therapy are still lacking. Disease progression can cause irreversible bone and joint damage. The prevalence of SAPHO syndrome was reported as 1/10,000 in Caucasians and 0.00144/100,000 in the Japanese population[6].

The most widely applied diagnostic criteria was formulated in 1988 by Benhamou et al, who established the diagnosis of SAPHO syndrome based on at least one of four criteria: (1) osteoarticular manifestations of acne conglobate, acne fulminans, or hidradenitis suppurativa; (2) osteoarticular manifestation of PPP; (3) hyperostosis (of the anterior chest wall, limbs or spine) with or without dermatosis; and (4) chronic recurrent multifocal osteomyelitis (CRMO) involving the axial or peripheral skeleton with or without dermatosis[7]. Another commonly used diagnostic criterion was proposed in 1994 by Kahn and Kahn, who established the diagnosis of SAPHO syndrome based mainly on pathological examinations[8].

SAPHO syndrome shares a variety of features with seronegative spondyloarthropathies (SpAs) such as ankylosing spondylitis (AS), psoriatic arthritis (PsA) and sacroiliitis[4]. Extra-articular manifestations have been widely reported in cases of seronegative polyarthritis, indicating the necessity of maintaining a high level of awareness of systemic involvement when treating both active seronegative polyarthritis and SAPHO syndrome[9]. Although the extra-articular manifestations of AS are well defined, the systemic involvements of SAPHO syndrome have only occasionally been reported[9]. Previous cases of concomitant pleural effusion [10,11] and organizing pneumonia [12] have been reported. However, to our knowledge, no study has evaluated pulmonary comorbidity in SAPHO syndrome.

In our clinical work, we have observed pulmonary lesions in some patients. However, the relationship between pulmonary manifestations and disease activity remains unclear. The aim of this study was to determine pulmonary manifestations in SAPHO syndrome patients and explore the association between disease activity and pulmonary manifestations based on reconstructed CT images.

## Materials and methods

The Institutional Review Board of the Peking Union Medical College Hospital (PUMCH) approved this study (research ethics board approval number: ZS-944).

Pulmonary HRCT images were reconstructed from whole-spine CT images of SAPHO syndrome patients and healthy controls to evaluate pulmonary comorbidity and its correlation with disease activity in SAPHO syndrome.

The inclusion criteria for SAPHO syndrome patients in our study were based on the Benhamou criteria. The Benhamou criteria stipulate that the presence of 1 of the 4 inclusion criteria is sufficient to establish diagnosis. However, in this study, only those with osteoarticular manifestations with PPP were included to enroll a relatively homogeneous patient population. The inclusion criteria were as follows: (1) anterior chest wall involvement, diagnosed by ^99 m^Tc-methylene diphosphonate (^99 m^Tc-MDP) bony scintigraphy; (2) characteristic PPP as the only cutaneous manifestation; and (3) a history of or current pain on at least one spinal level. From December 2014 to February 2016, a total of 69 SAPHO syndrome patients met the criteria and were enrolled. Whole-spine computed tomography (CT) examinations using the Toshiba Aquillion ONE 320-detector CT were prospectively performed at the Department of Radiology at PUMCH for standard disease evaluation. Relevant examination protocols were employed as follows: tube voltage 120 kV, thickness 2 mm, window width 2000 Hounsfield units (HU), and window level 400 HU. Chest reconstructions (from the lung apices to the costophrenic sinus) were obtained using the following protocol: thickness 2 mm, window width 1200 HU, and window level -600 HU.

Demographic data and clinical features, including a disease activity evaluation, were recorded. Laboratory evaluations, including erythrocyte sedimentation rate (ESR) and highly sensitive C-reactive protein (hs-CRP), were measured within 3 days of the CT examination. The reference ranges for ESR and serum hs-CRP were adjusted by age and gender. The age-adjusted approximate upper reference limit (mm/h) for ESR was ESR = age/2 for males and ESR = (age+10)/2 for females[13]. The age-adjusted approximate upper reference limit (mg/dl) for hs-CRP was hs-CRP = age/50 for males and hs-CRP = age/50+0.6 for females[14]. Among the SAPHO syndrome patients, 67 had reconstructed pulmonary HRCT images available and were included in our study. We evaluated the disease activity of the SAPHO syndrome patients with the Bath Ankylosing Spondylitis Disease Activity Index (BASDAI). The BASDAI is a standard instrument that is regularly used to assess disease activity in AS patients [15]. A BASDAI score of at least 4.0 (out of a maximum of 10) indicates active disease. The patients were divided into an active group (BASDAI ≥4) and a stable group (BASDAI <4).

The healthy control group comprised 58 volunteers who participated in an environmental cohort study at PUMCH from June 2016 to July 2017 (clinical trial No. NCT03193879). All the participants were adults who younger than 70 years of age and were confirmed to be healthy; rheumatic diseases and respiratory illnesses were ruled out after screening by medical history inquiry, routine blood tests, ESR, hs-CRP, pulmonary function test and chest HRCT scan. In the healthy control group, chest HRCT examinations were performed using a 128-section dual-energy CT system (SOMATOM Definition Flash, Siemens Healthcare, Forchheim, Germany). The following examination protocols were applied: tube voltage 120 kV, thickness 2 mm, window width 1200 HU, and window level -600 HU. The scan ranged from the costophrenic angle to the pulmonary apex. The participants were instructed to take a deep breath and hold it during the CT examination. The ESR data of seven patients were unavailable, while the hs-CRP data were missing for twenty-seven patients. The reference ranges for ESR and serum hs-CRP were adjusted by age and gender as described above.

At least one radiologist and one pulmonologist reviewed the HRCT images for each case. Computed tomography images were retrieved by entering the patient ID into our electronic hospital information system. In this way, the observers were blind to the subjects’ characteristics. The observers independently documented abnormal findings. Discrepancies were presented to and verified by a third expert.

The following abnormalities were documented and analyzed: nodules (solitary/multiple) in the lung, opacities, consolidation, cavities, masses, ground-glass opacity, reticular patterns, irregular linear opacity, bronchiectasis, bronchial wall thickening, tree-in-bud sign, emphysema, pulmonary bulla, cysts, pleural thickenings, lymph node calcification, and additional findings. The HRCT patterns are defined in S1 Table.

All statistical analyses were performed using a personal computer and SPSS 23.0 (SPSS, Inc., USA) software. Metric variables are expressed as either the median plus interquartile range [IQR] for nonparametric data or the mean ± standard deviation [SD] for parametric data. Categorical data are summarized as the percentage of the total group. Differences in quantitative data distribution between patient subgroups were compared using Student’s t test for parametric data and the Mann-Whitney rank-sum test for nonparametric data. Differences in the frequencies of categorical data were compared using the χ2 test. A *p* value < 0.05 was considered statistically significant. We performed repeated multivariate logistic regression. Full-model multivariate logistic regression analysis was used to determine the value of gender, age, disease duration, ESR level, CRP level, and BASDAI for predicting abnormal pulmonary findings on HRCT.

## Results

Patient demographic information is shown in Table 1.

**Table 1:** Demographic features of the SAPHO syndrome patients and healthy controls.

Parameter	SAPHO patients (n = 67)	Healthy controls (n = 58)	*p* value
Age (year)			
Mean±SD/median [IQR]	47.0[38.0–53.0]	37.0[30.8–53.8]	0.022
Gender			
Female	54(80.6%)	36(53.7%)	0.021
Male	13(19.4%)	22(32.8%)	
Onset age (years)			
Mean±SD	42.1±9.7		
Age of initial cutaneous symptoms (years)			
Mean±SD	42.6±9.8		
Age of initial osteoarticular symptoms (years)			
Mean±SD	42.9±9.7		
Duration (months)			
Median [IQR]	20.0[8.0–36.0]		
BASDAI			
≤4.0	32(47.8%)		
>4.0	35(52.2%)		
ESR (mm/h)			
Male	(n = 13) 23.0[12.0–45.5]	(n = 21) 2.0[2.0–5.0]	*p*<0.001
Female	(n = 54) 29.0[14.0–53.8]	(n = 30) 8.5[6.8–14.3]	*p*<0.001
hs-CRP (mg/L)			
Male	(n = 13) 19.5[7.7–62.4]	(n = 8) 1.0[0.5–2.8]	0.001
Female	(n = 54) 8.6[2.2–18.0]	(n = 24) 0.6[0.3–1.4]	*p*<0.001

According to the detailed HRCT evaluations, the overall rate of pulmonary abnormalities in the SAPHO syndrome patients was 67.2% (45 of 67 patients), which was similar to that of the healthy controls (39 of 59 patients, 67.24%). Compared to the healthy controls, the SAPHO syndrome patients had a significantly higher rate of patchy shadows (*p* = 0.037) but a significantly lower percentage of nodules (*p* = 0.006), especially multiple nodules (*p* = 0.001). Table 2 shows the abnormal HRCT findings that were detected in the SAPHO syndrome patients and healthy controls.

**Table 2:** Distribution of abnormal HRCT findings in SAPHO syndrome patients and healthy controls.

Abnormalities	SAPHO patients		Healthy controls		*p* value
	n	Percentage (%)	n	Percentage (%)	
Abnormal HRCT findings	45	67.2	39	67.2	0.993
Irregular linear opacity	29	43.3	22	37.9	0.544
Opacity	22	32.8	9	15.5	0.025
Ground-glass opacity	11	16.4	11	19.0	0.709
Pleural thickening	9	13.4	10	17.2	0.554
Nodules in lung	7	10.4	18	31.0	0.004
Solitary nodule in lung	6	9.0	6	10.3	0.793
Multiple nodules in lung	1	1.5	12	20.7	p<0.001
Bronchiectasis	3	4.5	0	0	0.248
Pulmonary bulla	2	3.0	4	6.8	0.415
Reticular pattern	1	1.5	0	0	1.000
Emphysema	1	0	4	6.9	0.182
Calcification of lymph nodes	0	0	2	3.4	0.213

**Fig 1:**
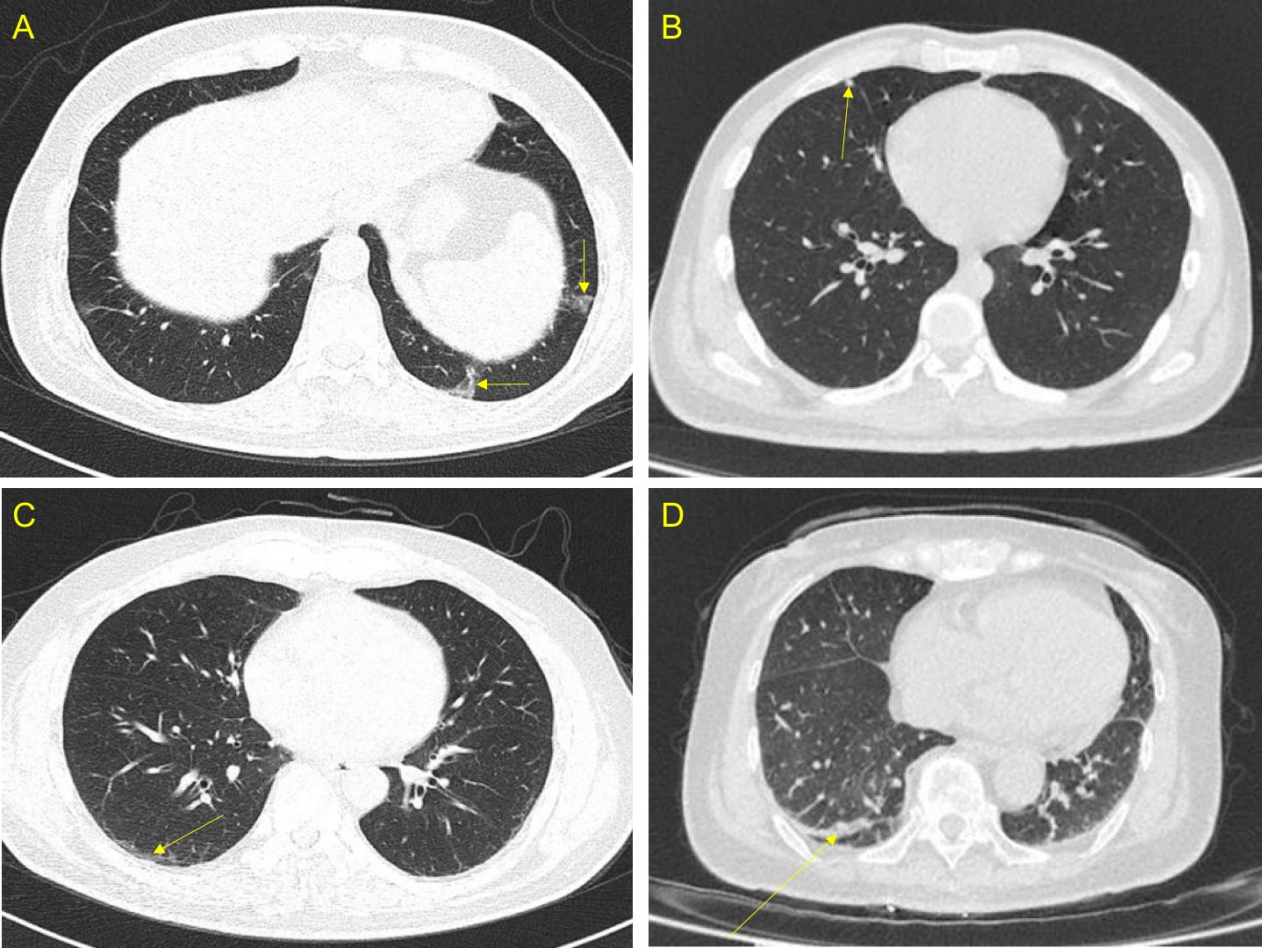
High-resolution computed tomography images. A: Ground-glass opacity (arrow); B: Solitary nodule (arrow); C: Stripe (arrow), and D: Patchy shadows and stripe (arrow).

Typical HRCT findings are presented in Fig 1.

The age, gender, onset age, duration, BASDAI, ESR, and hs-CRP distributions of the SAPHO patients with and without abnormal HRCT findings are shown in Table 3. Multivariate logistic regression analysis was employed to identify possible predictors of abnormal HRCT pulmonary findings in the SAPHO syndrome patients (Table 4).

**Table 3:** Distribution of the clinical characteristics of SAPHO syndrome patients with and without abnormal HRCT findings.

Abnormalities	Abnormal HRCT findings (n = 45)	No abnormal HRCT findings (n = 22)	*p* value
Age			
	51.0[45.5–54.5]	38.3±7.9	*p<0*.*001*
Gender			
Male	9	4	1.000
Female	36	18	
Onset age (years)			
	45.7±8.4	34.9±7.9	*p<0*.*001*
Duration (years)			
	1.5[0.7–3.8]	1.9[0.7–3.5]	0.602
BASDAI			
≤4.0	23	9	0.432
>4.0	22	13	
ESR (mm/h)			
Male	23.0[10.5–55.0]	20.0[12.3–28.5]	0.710
Female	30.0[14.8–67.8]	22.0[14.0–41.5]	0.393
hs-CRP (mg/L)			
Male	19.5[4.6–62.4]	23.3[12.2–77.1]	0.604
Female	10.8[3.4–22.9]	3.3[1.4–10.9]	0.040

**Table 4:** Predictors of abnormal pulmonary HRCT findings in SAPHO syndrome patients (multivariate logistic regression).

	Multivariate logistic regression results	
	OR[95% CI]	*p* value
**Gender**		
Female vs male	0.466[0.083,2.623]	0.387
**Age**		
Per year	1.267[1.123,1.430]	*P<*0.001
**Duration**		
Per year	0.965[0.817,1.139]	0.671
**BASDAI**		
≥4 VS <4	0.116[0.017,0.809]	0.030
**CRP**		
Above normal vs normal	3.165[0.390,25.707]	0.281
**ESR**		
Above normal vs normal	0.625[0.148,2.644]	0.523

Multivariate logistic regression analysis was also applied to both the SAPHO syndrome patients and the controls (Table 5). Age was the only significant predictor of pulmonary HRCT abnormalities.

**Table 5:** Multivariate logistic regression of abnormal pulmonary HRCT findings in SAPHO syndrome patients and healthy controls.

	Multivariate logistic regression results	
	OR[95% CI]	*p* value
**Gender**		
Female vs male	0.441[0.111,1.756]	0.246
**Age**		
Per year	1.135[1.063,1.212]	*P<*0.001
**CRP**		
Above normal vs normal	0.633[0.149,2.681]	0.534
**ESR**		
Above normal vs normal	0.456[0.133,1.563]	0.212

## Discussion

This study is the first to perform a comprehensive analysis of pulmonary comorbidities in SAPHO syndrome. HRCT was employed for detailed examinations since it can show minor changes much earlier than chest X-ray. Of the 67 patients included in the present study, 45 (67.2%) had abnormal pulmonary findings. The most common findings were irregular linear opacities (29, 43.3%), opacities (22, 32.8%) and ground-glass opacity (11, 16.4%).

Pulmonary involvement in rheumatic diseases is a common manifestation and a major cause of mortality and morbidity. Therefore, the early diagnosis and effective treatment of pulmonary comorbidities is of paramount importance to optimize management[16]. In SpAs, 40–85% of patients have lung involvement according to HRCT studies[17]. Furthermore, recent studies have revealed a higher prevalence of pulmonary lesions in AS than previously thought[18]. A previous study suggested that the pulmonary involvement in SpAs was caused by an unspecified immunological process[19]. Pulmonary lymphocyte alveolitis was reported as a subclinical disease in SpAs[20]. Since SAPHO syndrome shares a variety of features with SpAs, it is also necessary to guarantee the early evaluation of pulmonary comorbidities and the proper management of potential pulmonary lesions.

Previous case reports have discussed possible associations between pulmonary abnormalities and SAPHO syndrome. In 1995, Vaile et al reported a case with multiple necrotic pulmonary nodules[21]. In 2001, Fernandez-Campillo et al reported the case of a 61-year-old man who presented with scalp psoriasis, pubic osteitis, pain and stiffness of the anterior chest wall, and right exudative pleural effusion[10]. In 2017, Hasegawa et al described the case of a 66-year-old woman who had SAPHO syndrome with marked sternal osteitis and bilateral pleural effusions[11]. Additionally, in 2017, Hameed et al presented the case of a 57-year-old woman with SAPHO syndrome complicated by organizing pneumonia (OP) that manifested as recurrent episodes of pneumonia[12]. Increased uptake of ^18^F-FDG was also observed in some SAPHO syndrome patients in our previous study (unpublished). However, studies evaluating lung involvement in SAPHO syndrome have not been previously conducted.

Although the overall rates of abnormal HRCT findings in the SAPHO syndrome patients and the reference group were similar, the distribution of abnormal findings differed between the two groups. Compared with the reference group, the SAPHO syndrome patients had a significantly higher rate of opacities (*p* = 0.037) but a significantly lower percentage of nodules (*p* = 0.006), especially multiple nodules (*p* = 0.001). Opacities indicate the existence of inflammation, which may be the pulmonary manifestation of a systemic inflammatory state that also presents as inflammatory osteoarticular symptoms and skin changes. The major causes of pulmonary complications in rheumatic diseases should be considered in the pulmonary comorbidities of SAPHO syndrome, which include interstitial pneumonia, vasculitis and pleuritic[22].

The lower incidence of pulmonary nodules, especially multiple nodules, in the SAPHO syndrome patients may be partly explained by the interference of patchy shadows. Pulmonary nodules may be more difficult to detect when they are located within or in the vicinity of a patchy shadow. Since the size of nodules was not determined in the image analysis, the significance of the nodules in the control group may be overestimated. Lung nodules could be detected at a certain frequency in healthy population. For instance, Gould et al examined 415,581 chest CT examinations of more than 200,000 adults and estimated the frequency of nodule identification in the U.S. population to be 24–31%[23]. Besides, the absence of abnormalities such as opacities and consolidation make in the control group may make the nodules more prominent.

Multivariate logistic regression was performed to examine whether BASDAI, ESR, CRP, disease duration, gender and age were possible predictors of abnormal HRCT pulmonary findings. Since hs-CRP data were missing for 27 patients, hs-CRP could not be included in the logistic regression analysis. Age was found to be the strongest predictor, indicating an association between older age and a higher rate of abnormal HRCT pulmonary findings. BASDAI was another significant predictor, suggesting that SAPHO syndrome patients with BASDAIs less than 4 are more likely to have pulmonary manifestations on HRCT. These results suggest that a high level of awareness of pulmonary abnormalities should be maintained when treating SAPHO syndrome patients who are older and have a BASDAI < 4. Gender and other inflammatory indicators were not significant predictors of abnormal HRCT pulmonary findings according to our analyses.

In this study, bronchial wall thickening and tree-in-bud patterns were not observed in the SAPHO syndrome patients, indicating that the pulmonary comorbidities of SAPHO syndrome may not involve the small airways. Consolidation and cavitation were also not identified, suggesting that SAPHO-associated pulmonary manifestations may be less severe. However, these observations must be confirmed in a larger sample.

There are several limitations of the present study. First, smoking habit data were not collected from the SAPHO group. Since abnormalities related to smoking, such as emphysema and bullae, did not differ between the SAPHO syndrome patients and the control group, smoking habits would likely not have had much influence on the conclusion. Second, the SAPHO syndrome patients included in this study were not comparable with the healthy controls. Third, fewer than 70 patients were included, although this is not a small number considering the rarity of SAPHO syndrome. Fourth, the lack of long-term follow-up makes it difficult to further explain the correlation between SAPHO syndrome and pulmonary manifestations. Finally, pathological explanations were unavailable because the patients were unwilling to undergo biopsy.

## Conclusions

In this study, we first explored the pulmonary changes in SAPHO syndrome patients by detailed examination using HRCT. Although the overall rates of abnormal pulmonary manifestations were similar between the SAPHO syndrome patients and the reference group, the distribution of abnormal findings differed between the two groups: the SAPHO syndrome patients had a significantly higher rate of opacities and a significantly lower percentage of nodules than the healthy controls. In addition, disease activity appeared to be associated with the appearance of pulmonary lesions. Therefore, awareness of pulmonary abnormalities should be maintained when treating SAPHO syndrome patients with an older age and a BASDAI < 4.

## Supporting information

S1 Table Definitions of HRCT patterns. (DOCX)

S2 Table Demographic features, laboratory data and HRCT findings of the healthy controls. (XLSX)

S3 Table Demographic features, laboratory data and HRCT findings of the SAPHO patients. (XLSX)

S1 File STROBE checklist. (DOCX)
