# Supplementary material for: Pulmonary high-resolution computed tomography findings in patients with synovitis, acne, pustulosis, hyperostosis and osteitis syndrome
Source: PLoS One. 2018 Dec 5;13(12):e0206858. doi: 10.1371/journal.pone.0206858 (PMC6281176; doi:10.1371/journal.pone.0206858)
Supplement: S1 Table — (DOCX) [file pone.0206858.s001.docx]

**Supplementary Table 1. Definitions of HRCT patterns.**

| Term | Definition |
| --- | --- |
| Nodule (solitary/multiple) | A rounded opacity, well or poorly defined, measuring up to 3 cm in diameter |
| Opacity | Any area that preferentially attenuates the X-ray beam and therefore appears more opaque than the surrounding area |
| Consolidation | A homogeneous increase in pulmonary parenchymal attenuation that obscures the margins of vessels and airway walls |
| Cavity | A gas-filled space, seen as a lucency or low-attenuation area, within pulmonary consolidation, a mass, or a nodule |
| Mass | Any pulmonary, pleural, or mediastinal lesion seen on chest radiographs as an opacity greater than 3 cm in diameter (without regard to contour, border, or density characteristics) |
| Ground-glass opacity | A hazy increased opacity of lung, with preservation of bronchial and vascular margins |
| Reticular pattern | A collection of innumerable small linear opacities that, by summation, produce an appearance resembling a net |
| Irregular linear opacity | Irregular, linear shaped opacities more than 5 mm in diameter |
| Bronchiectasis | Bronchial dilatation with respect to the accompanying pulmonary artery (signet ring sign), lack of tapering of bronchi, and identification of bronchi within 1 cm of the pleural surface |
| Bronchial wall thickening | The bronchial walls appear thick and usually less well-defined than normal |
| Tree-in-bud | Centrilobular branching structures that resemble a budding tree which reflects a spectrum of endo- and peribronchiolar disorders, including mucoid impaction, inflammation, and/or fibrosis |
| Emphysema | Focal areas or regions of low attenuation, usually without visible walls |
| Pulmonary bulla | A rounded focal lucency or area of decreased attenuation, 1 cm or more in diameter, bounded by a thin wall |
| Cyst | A round parenchymal lucency or low-attenuating area with a well-defined interface with normal lung |

(The definitions were cited from *David M Hansell DAL, H Page McAdams, Alexander A Bankier. Imaging of diseases of the chest. 5th ed. Philadelphia: Elsevier/Mosby; 2010.*)
